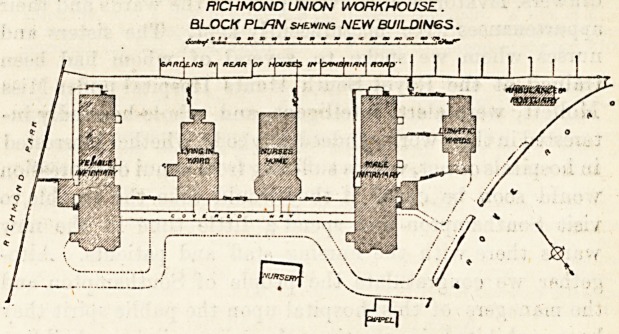# Richmond Union Infirmary

**Published:** 1901-08-31

**Authors:** 


### The Institutional Workshop.

### RICHMOND UNION INFIRMARY.

The foundation-stone of these buildings was laid in May, 1901. There are four blocks connected by a three-storey corridor, and a fifth and sixth block entirely detached; in the latter are placed the mortuary and the ambulance room; and the former contains the lying-in wards and dispensary. This connecting corridor is an important feature in the plan,

RICHMOND UNION WORKHOUSE.
PROPOSED MALE INFIRMARY.

FIRST FLOOR PLAN.

for by it communication is obtained between the various floors of the large infirmary blocks, and the lying-in wards and the Nurses' Home are also reached from it by short corridors. The difficulty of supervision inseparable from a large building in detached blocks is thus very materially lessened.

The female infirmary is placed at the eastern end of the main corridor and is three stories high, each floor giving accommodation for 27 beds. There are two dormitories, of which the larger for 24 beds is placed southward of the corridor, and the smaller for three beds is placed northwards. The larger is about 70 feet long by 23 feet wide, and assuming it to be 12 feet high each patient would have a little over 800 cubic feet of space and 67 feet of floor space. For a general infirmary this allowance would be considered insufficient, but not for a union infirmary, as the cases usually under treatment in the latter differ materially from those in the former. After all that has been said about floor and cubic space in any sick wards, we are convinced that more depends on the efficiency with which the ventilation is

GROUND FLOOR PLAN.

carried out; but with the smaller floor spaces it is practically impossible to obtain a window on both sides of each bed. Here each pair of beds has the windows so disposed, and, perhaps, in a union infirmary that is as much as we have any right to expect. Adjoining the dormitory is a nurses' duty-room, then the hall with the main staircase and a door to the corridor and the three-bedded ward. This ward has a bay window of large size in the end, and there is another window in the side of the room, but there is no proper cross-ventilation, and what is of more importance the sanitary block is not cut off by a ventilating passage. This omission is rather curious, as this essential point has been attended to in the larger sanitary block attached to the large ward. A day-room occupies the other corner, and there are storeroom, linen-room, larder and foul-air extraction shafts.

A terrace surrounds the large ward on two sides, and there is an external fire escape staircase. The first and second floors are similar in details excepting that balconies take the place of the terrace. These terraces and balconies are most useful adjuncts to an infirmary.

The male infirmary is placed at the other end of the corridor, and is practically the same as the female one just described. Between these are the lying-in ward and the nurses' home. The former is a one-storey block and the latter consists of three stories.

The fifth block is for lunatics. It is of one storey only, and is intended for temporary residence of the patients. There are two two-bedded rooms and a padded room, with attendants' rooms and the proper offices.

The work is to be done in bricks of a rich deep red colour relieved by strings of Portland stone. The roofs will be covered with green slates.

The ground floor wards will be laid in pitch pine blocks and the corridors in terrazzo. The sanitary spurs will be

RICHMOND UNION WORKHOUSE.
BLOCK PLAN SHEWING NEW BUILDINGS.

lined with glazed tiles. The ward walls will be plastered with hard cement we hope, and the angles and corners of the rooms are rounded off.

Very properly the warming is to be carried out with open fires, and hot water radiators are placed under some of the windows. Foul air will be extracted by aluminium fans.

Fire hydrants are placed on all the floors.

The surface water and the sewage are conveyed in separate channels.

The architect is Mr. Edward J. Partridge, F.S.I., of George Street, Richmond.

The contract for the whole building as at present designed was £33,800; but this will be somewhat exceeded as the Guardians contemplate the erection of another block for the open-air treatment of consumption.

## Figures and Tables

**Figure f1:**
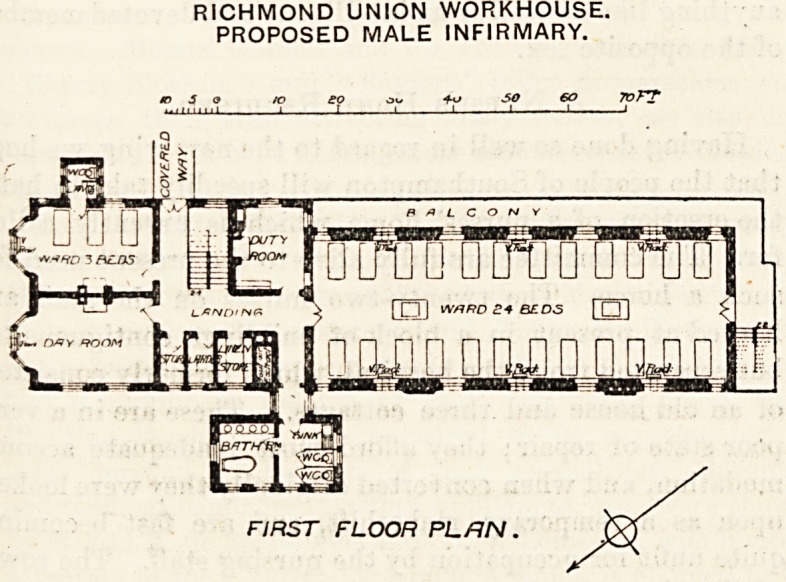


**Figure f2:**
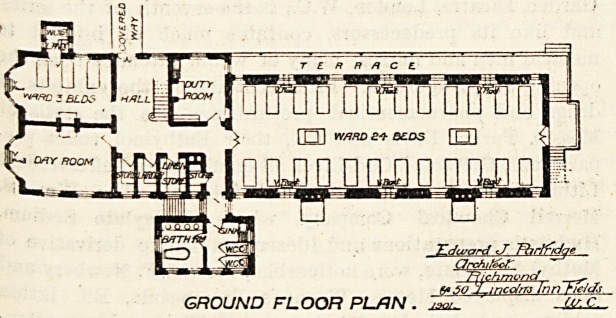


**Figure f3:**